# From the MMC Specification to Endosperm Cellularization in Arabidopsis: A Developmental-Handover Framework for Seed Initiation

**DOI:** 10.3390/plants15091410

**Published:** 2026-05-05

**Authors:** Prakash Babu Adhikari, Ryushiro Dora Kasahara

**Affiliations:** Bioscience and Biotechnology Center, Nagoya University, Nagoya 466-8560, Japan; kasahara.ryushiro.s2@f.mail.nagoya-u.ac.jp

**Keywords:** megaspore mother cell, female gametophyte, central cell, Polycomb Repressive Complex 2, auxin, genomic imprinting, syncytial endosperm, endosperm cellularization

## Abstract

Seed initiation in Arabidopsis depends on regulatory transitions that begin before fertilization, yet these events are often treated as separate developmental episodes rather than as a connected sequence. Here, we synthesize evidence from megaspore mother cell (MMC) specification to endosperm cellularization and ask whether particular stage boundaries meet a narrow definition of developmental handover: a shift between dominant control logics, with detectable first-order consequences in the ensuing interval and acknowledged overlap across the boundary. This framework goes beyond canonical staging by distinguishing chronological succession from shifts in regulatory control, thereby clarifying where earlier states are expected to constrain later outcomes, which developmental boundaries are mechanistically well supported, and where further mechanistic resolution is most needed. We first examine how MMC singleness (restriction to a single reproductive founder cell per ovule primordium) emerges through coupled sporophytic restriction and local competence. We then consider how meiosis and female gametophyte maturation establish regulatory poise (an actively restrained and asymmetric mature female-gametophytic state), including cell-cycle restraint, companion-cell-restricted demethylation, and unequal gametic chromatin states that condition subsequent embryo and endosperm behavior. After fertilization, release of central-cell restraint, activation of an endosperm auxin program, and recruitment of maternal tissues together mark the onset of seed initiation. In this view, syncytial endosperm is an actively maintained developmental state shaped by parental dosage, epigenetic control, hormone signaling, and maternal interaction, whereas endosperm cellularization represents a regulated switch with seed-wide consequences. In Arabidopsis, the clearest handover is the mature female gametophyte-to-fertilization boundary, whereas the boundaries linking MMC specification to female gametophyte maturation and syncytial endosperm to cellularization remain provisional.

## 1. Introduction

Early seed development in flowering plants is often described as a sequence of discrete events: specification of the megaspore mother cell (MMC), meiosis and female gametophyte formation, double fertilization, syncytial endosperm proliferation, and endosperm cellularization. However, that chronology does not by itself distinguish stage succession from a shift in developmental control. Across early seed initiation, successive transitions are shaped by changes in what constrains developmental competence, where that control is imposed, and how it is relieved. This is already evident before fertilization, when reproductive fate is specified within a somatic ovule context rather than in isolation [1,2,3,4,5]. It remains evident after fertilization, when the embryo, endosperm, and maternal tissues exchange signals that shape seed initiation and early development, rather than progressing independently [6,7,8,9,10,11,12]. Yet much of the literature remains organized around one stage at a time, so the transfer of dominant control logic between successive intervals is more often implied than explicitly tested. Endosperm cellularization likewise marks a major developmental transition in many angiosperms, although its timing, extent, and morphology vary substantially across lineages [13,14,15,16].

Here, we ask whether the interval from MMC specification to the onset of endosperm cellularization can be read as a sequence of developmental handovers. We use handover more narrowly than generic transition language. A proposed boundary qualifies as a developmental handover only when four conditions can be assessed: (1) the dominant control logic organizing or constraining progression before the boundary is identifiable; (2) a distinct dominant control logic organizing or constraining progression after the boundary is identifiable; (3) crossing the boundary has detectable first-order consequences in the ensuing interval; and (4) earlier and later control logics or influences remain partially overlapping across the boundary rather than switching instantaneously. In this usage, a regime is not a single regulator or a whole cell state by itself, but the dominant interval-level restraining or organizing logic inferred from the relevant cellular state, developmental constraints, and best-supported molecular modules. Throughout, restraint denotes active restriction of developmental or proliferative competence rather than mere developmental inactivity. Table 1 summarizes the developmental roadmap from MMC specification to endosperm cellularization, and Table 2 applies this criterion-based assessment boundary by boundary. “Established handover” denotes transitions for which all four conditions are directly supported in Arabidopsis. “Provisional handover” denotes transitions for which a shift in dominant control is biologically plausible and partly supported, but one or more elements, most often dominance, ordering, or overlap, remain partly inferential.

Throughout this review, boundaries refer to stage changes expressed as X→Y, whereas intervals denote the developmental spans between those boundaries. Labels such as licensing and syncytial maintenance therefore name interval-level regimes rather than boundaries themselves. Boundary-level evidence asks whether a change in dominant control can be localized and justified at a particular developmental juncture; interval-level evidence asks how a given state is maintained, stabilized, or propagated once that juncture has been crossed. A later interval may therefore be mechanistically well characterized without, on its own, proving that the boundary leading into it represents a resolved handover.

Because the underlying biology is not resolved at a single temporal scale, the assessed boundaries are not uniform in duration. Some are event-centered, such as fertilization, whereas others are longer composite boundaries in which the change in dominant control is distributed across a broader developmental span. In those cases, the unit under assessment is not the full interval, but the proposed shift in developmental control that links one interval to the next. Boundaries were therefore downgraded whenever criterion 2 depended on several equally plausible organizers, or criterion 3 could be supported only by general features of the later interval rather than by clearly attributable first-order consequences of crossing the boundary.

Arabidopsis provides the strongest basis for such a synthesis because early reproductive development has been dissected there with exceptional genetic, epigenetic, and cytological resolution. Accordingly, this review remains anchored in Arabidopsis unless stated otherwise. Comparisons with other angiosperms are introduced selectively to clarify which features of the proposed handover logic appear broadly relevant, which represent recurring developmental problems without a single shared mechanism, and which remain contingent on lineage, endosperm type, or developmental context rather than implying uniform conservation.

A reappraisal along these lines is timely for two reasons. First, MMC singleness is no longer satisfactorily explained by single-gene or strictly cell-autonomous models. Instead, current evidence supports a multilevel view in which small-RNA pathways, DNA methylation balance, chromatin remodeling, ovule identity factors, and localized auxin responsiveness together delimit one reproductive founder cell within the nucellus [1,2,3,4,17,18]. Second, post-fertilization development is increasingly resolved as a distributed process in which fertilization-triggered release, paternal inputs, endosperm-autonomous hormonal programs, and feedback from maternal tissues jointly shape the conditions under which endosperm proliferation and embryogenesis proceed [6,7,8,9,10,11,12]. These advances make it possible to ask a more precise question than chronology alone allows: where does developmental control appear to change hands, which first-order consequences become detectable in the ensuing interval, and where does the evidence remain too diffuse to support a strong boundary-level claim?

**Figure 1:**
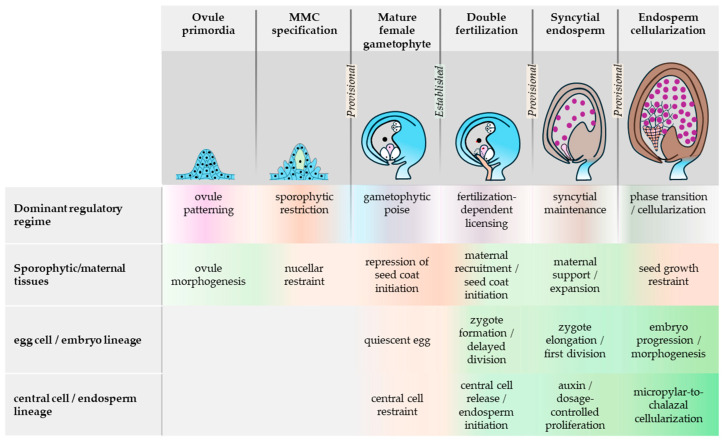
**Qualitative roadmap of early Arabidopsis seed initiation from ovule primordium development to endosperm cellularization.** Columns indicate developmental reference stages, and the dominant control logic row summarizes the principal organizing or restraining logic proposed for each interval. Labels at selected stage boundaries indicate boundary status under the manuscript’s handover criteria (Table 1; boundary-by-boundary assessment in Table 2). **Established** denotes a boundary directly supported in Arabidopsis, whereas **Provisional** denotes a biologically plausible shift in dominant control for which one or more elements, most often dominance, ordering, or overlap, remain partly inferential. Fertilization-triggered release of central-cell restraint is treated as the clearest established handover; the ensuing syncytial interval is shown more conservatively as a strongly supported maintained program whose separation from the preceding licensing transition remains partly overlapping. Overlap across rows indicates continuity and partial coexistence of influences rather than abrupt replacement. In the bottom three rows, orange shading indicates relatively restraining or repressive influence, green shading indicates relatively permissive or activating influence, and deeper shading indicates greater schematic emphasis within an interval rather than quantitative magnitude. Gray regions indicate processes not emphasized in this schematic. The figure is interpretive and qualitative and does not encode quantitative timing, effect size, or complete pathway resolution.

Therefore, the sections that follow pursue a deliberately limited argument. We do not propose a universal master regulator linking ovule patterning, gametic quiescence, fertilization, syncytial proliferation, and cellularization. Instead, we ask which stage boundaries satisfy the criteria for an established handover and which remain provisional, while also noting where similar regulatory logic recurs across distinct developmental contexts without warranting a separate boundary-level classification. The resulting map is intentionally uneven. In the current Arabidopsis evidence base, the fertilization-triggered release of central-cell restraint is the clearest established handover. Entry into a maintained syncytial endosperm program is biologically well supported but is treated here as the ensuing interval and as a conservative, provisional assessment case rather than as a second equally independent established handover. The transitions flanking MMC singleness and syncytial exit remain more provisional, and the developmental consequences of cellularization are better framed as heterogeneous downstream effects than as a further handover in their own right. The value of this framework lies in forcing explicit comparison between chronological succession and shifts in regulatory control, thereby clarifying which boundary consequences emerge, which states are reset or reconfigured, and where overlap between successive control logics is strongest or weakest across early seed initiation (Table 1; Figure 1).

## 2. Restricting Reproductive Fate: How MMC Singleness Emerges

**Figure 2:**
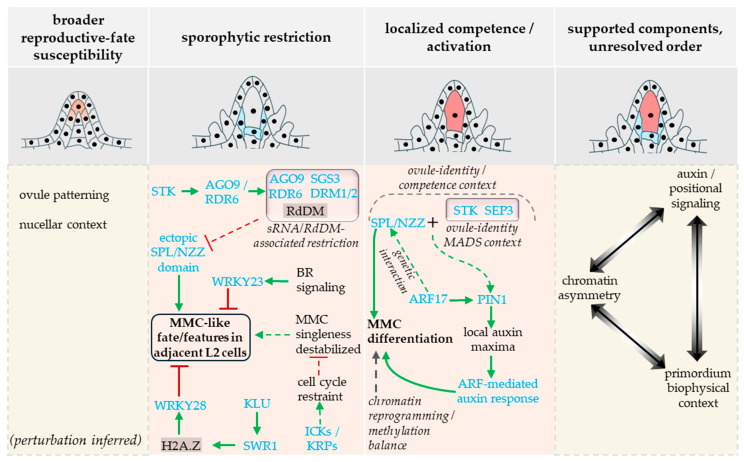
**Evidence-calibrated schematic of MMC singleness in Arabidopsis.** MMC singleness is interpreted here as the outcome of distributed sporophytic restriction together with localized competence within the ovule primordium, rather than as evidence for a uniform pre-patterned competence field. The left panel indicates broader reproductive-fate susceptibility inferred from perturbation phenotypes and expressed as graded or variable acquisition of MMC-like fate or features. The restriction arm summarizes supported Arabidopsis modules that limit supernumerary MMC-like fate or features in adjacent L2 cells. The competence arm summarizes supported Arabidopsis factors promoting MMC differentiation. The final panel indicates that the relative ordering among chromatin asymmetry, auxin/positional signaling, and primordium biophysical context remains unresolved, even though all three are supported components of the broader problem. Solid colored edges indicate directly supported Arabidopsis interactions or dependencies; dashed colored edges indicate supported module placement without resolved ordering; dotted gray elements indicate contextual synthesis; black bidirectional connectors indicate supported components whose relative ordering remains unresolved; and dashed panel borders mark interpretive framing or unresolved architecture. The figure is qualitative and interpretive and does not imply a single ordered hierarchy for MMC specification.

In Arabidopsis, early female germline development is better described as a progressive restriction process operating across a small apical subepidermal domain than as the de novo appearance of an intrinsically unique founder cell. Quantitative 3D analysis of ovule primordium development shows that SMC/MMC-like characteristics can initially arise in more than one subepidermal cell and are then progressively restricted to a single reproductive cell during organ growth; altered primordium geometry delays that resolution, supporting a canalization model for singleness rather than instantaneous one-cell specification [19]. Natural-accession, hybrid, and ploidy-dependent variation in ectopic female gamete precursor formation further indicates that this restriction process is developmentally plastic and sensitive to epigenetic pathway activity, including *ARGONAUTE9* (*AGO9*)/*RNA-dependent RNA polymerase 6* (*RDR6*)-linked control [4,20]. Mutant phenotypes affecting small-RNA pathways, DNA methylation homeostasis, chromatin regulation, or positional signaling [3,4,17,18,20,21] argue against a fixed-selector model of MMC singleness. Instead, they indicate that reproductive fate is progressively delimited and stabilized within a broader sporophytic context. Since these perturbations are not phenotypically equivalent, the evidence for broadened reproductive competence should still be graded rather than treated as binary: some mutant genotypes generate enlarged nucellar cells only; others exhibit MMC markers; and only a subset proceeds into meiosis, functional megaspore formation, or later gametophytic development. Wild-type singleness is therefore best understood as the resolved output of a canalized restriction process rather than as evidence for a fully ordered causal hierarchy. An evidence-graded view of MMC singleness, separating sporophytic restriction, local competence, and later stabilization, is summarized in Figure 2.

One of the clearest lines of evidence for non-cell-autonomous restriction comes from *AGO9*-associated small-RNA pathways. Olmedo-Monfil et al. [4] showed that *AGO9* is specifically expressed in the cytoplasmic foci of the somatic companion cells of the L1 layer and, together with *RDR6*- and *SGS3*-dependent functions, acts to restrict ectopic reproductive fate. Later analysis of natural variation additionally detected *AGO9* in some stage 1 ovules (ovules with well-defined proximal and distal axes with no integument initiation) and ecotypes, as well as a transient nuclear signal in the MMC or related accessory cells, indicating that the non-cell-autonomous model should be grounded in the functional site of *AGO9*-associated control rather than in its categorical absence [20]. Mendes et al. [3] sharpened this framework by showing that RNA-directed DNA methylation helps restrict *SPOROCYTELESS*/*NOZZLE* (*SPL*/*NZZ*) expression to a single cell and that *drm1 drm2* ovules develop ectopic enlarged MMC-like cells. Their demonstration that *SEEDSTICK* (*STK*) directly promotes *AGO9* and *RDR6* expression further connects ovule identity with epigenetic restriction of reproductive fate. A second small-RNA branch supports the same general conclusion more directly: Su et al. [22] showed that *tasi*R-ARF biogenesis is spatially restricted to the nucellar epidermis (L1 cells), with evidence for movement of *tasi*R-ARFs into underlying hypodermal (L2) cells, and that ectopic expression of *tasi*R-ARF-resistant *ARF3* (*ARF3m*) specifically in those hypodermal cells is sufficient to generate multiple MMC-like cells. MMC singleness can therefore be described with confidence as a sporophytically enforced outcome in which surrounding ovule tissues actively suppress ectopic reproductive fate in adjacent subepidermal cells. A related ARGONAUTE-linked role has also been reported in maize, where AGO104 accumulates in somatic cells surrounding the female meiocyte and contributes to somatic fate repression in the germ cell non-cell-autonomously, while *ago104* mutants show apomixis-like defects [23], suggesting that sporophytic small-RNA-associated control of female germline development may extend beyond Arabidopsis, even if the phenotypic output differs.

Maintenance of somatic cell identity of the MMC surrounding hypodermal cells is additionally contributed non-cell-autonomously by the inner integument primordia-expressed *KLUH*/*CYP78A5* (*KLU*), at least in part through SWR1-dependent H2A.Z deposition at the *WRKY28* locus. *WRKY28* is exclusively expressed in the hypodermal cells and represses those cells from entering a reproductive program [18]. A parallel route is hormonal and positional. In Arabidopsis, a distal nucellar BR microenvironment restricts female germline fate to a single subepidermal cell through BRI1- and BZR1-family factor-dependent transient activation of *WRKY23* [24]. More recent work further shows that EPFL ligands expressed in the ovule epidermis and ERECTA-family receptors function upstream of this branch: EPFL-ERf signaling promotes BRI1 and BZR1-family activity and enables BZR1-dependent activation of *NSN1*, a sporophytic factor required to prevent multiple cells achieving MMC fate [25]. These data justify treating EPFL-ERf-BZR1-NSN1 as one directly linked MMC-restrictive branch within a broader landscape of sporophytic control. However, it is yet not clear whether all restrictive inputs operate within a single ordered hierarchy.

The selected MMC also undergoes a pronounced somatic-to-reproductive cell-state transition. She et al. [26] showed that differentiating MMCs undergo large-scale chromatin reprogramming relative to surrounding nucellar cells, including chromatin decondensation, reduced heterochromatin, histone-variant turnover, and altered histone-modification states, consistent with acquisition of a distinct premeiotic chromatin state. These data do not yet resolve whether chromatin reprogramming is an initiating cause of singleness, a permissive condition for reproductive entry, or a consequence of successful selection, but they show that MMC specification involves active remodeling of cell state rather than passive release from repression. Jiang et al. [17] added a further layer by showing that both hypo- and hypermethylated states can produce enlarged MMC-like cells and that proper maintenance of a single MMC depends on methylation-demethylation balance rather than on high or low methylation alone. Their single-cell analysis, which places mCHH decline before *KNUCKLES* (*KNU*) expression in the precursor MMC, likewise supports a progressive epigenetic transition rather than a binary switch. At present, the epigenetic evidence is strongest for differential state formation and stabilization, but weaker for assigning first-cause status within the broader restriction process.

Therefore, positive specification needs to be considered alongside restrictive control. SPL/NZZ remains the key positive regulator of MMC differentiation, yet current evidence places it within a broader ovule-patterning and auxin-responsive competence network rather than as an isolated lineage determinant. SPL/NZZ has been shown to function as an adaptor-like transcriptional repressor that recruits TPL/TPR co-repressors and antagonizes CIN-like TCP factors during ovule development, providing a biochemical framework for megasporocyte differentiation [27]. Genetic data further place SPL/NZZ within an auxin-linked competence module: an miR160 target ARF17 promotes MMC specification through interaction with SPL/NZZ and helps delimit *PIN1* expression and the normal auxin maximum at the ovule apex, whereas altered auxin signaling produces supernumerary MMCs in an *ARF17*- and *SPL*/*NZZ*-dependent manner [2]. Cavalleri et al. [1] extend this model by showing that *SPL*/*NZZ* cooperates with ovule-identity MADS-domain complexes and controls a *PIN1*- and *ARF*-dependent downstream network required for MMC differentiation (Figure 2, local activation/competence arm). The case for localized competence is therefore strong, but the ordering between competence acquisition and surrounding-cell restriction remains unresolved.

Cell-cycle restraint appears to provide an additional enforcement layer during MMC specification. In the Arabidopsis *ick*/*krp* septuple mutant, supernumerary MMCs, multiple functional megaspores, multiple embryo sacs, and occasional twin embryos were observed, showing that cyclin-dependent kinase inhibitors contribute both to one-MMC-per-ovule control and to developmental linkage with later megaspore selection [21]. Because these phenotypes arise in a high-order mutant with broad developmental consequences, they are best interpreted as evidence that cell-cycle control helps stabilize singularity and limit developmental overexpansion.

In the boundary-level assessment, meiosis is not treated as a separately resolved handover. This is not primarily a matter of manuscript scope, but of evidentiary resolution: the interval from MMC singleness to later female-gametophytic poise spans meiotic entry, functional megaspore selection, and gametophytic maturation, and current evidence does not localize a single boundary-localized shift in dominant control across that developmental arc with the precision available for fertilization. Meiosis is therefore part of why this first assessed unit must be treated as a composite pre-fertilization boundary rather than as a short event-centered transition. For that reason, it should not be read as directly comparable to the fertilization-centered boundary in Table 2.

Overall, Arabidopsis evidence supports a modular model in which MMC singleness emerges from distributed sporophytic restriction, localized competence, and subsequent stabilization of reproductive state. However, the causal ordering of those modules and the point at which their combined effects become a boundary to the ensuing interval-level control logic remain unresolved. MMC specification may therefore be viewed as a plausible, narrower gate-like event within this broader pre-fertilization span, but not yet as an operationally resolved handover in its own right. By the criteria used here, MMC singleness is instead best treated as part of a provisional composite pre-fertilization boundary: the underlying biology is substantial, and detectable first-order consequences are evident in the ensuing female-gametophytic interval, but a distinct dominant control logic linking MMC restriction to later female-gametophytic poise is not yet directly resolved across meiosis and early gametophytic differentiation. This classification should therefore be read as contingent on current boundary-level resolution rather than as a fixed limit on future mechanistic integration.

## 3. Gametophytic Poise After Meiosis: Asymmetric Conditioning and Pre-Fertilization Restraint

For clarity, we use *poise* in a restricted sense to denote four partly separable features of the mature female gametophyte: enforced cell-cycle restraint, unequal chromatin and transcriptional states in egg cell and central cell, central-cell-biased imprint-setting reprogramming, and residual intercellular communication before fertilization. These processes are related but not identical, and the section below treats them separately before arguing for their functional convergence.

**Figure 3:**
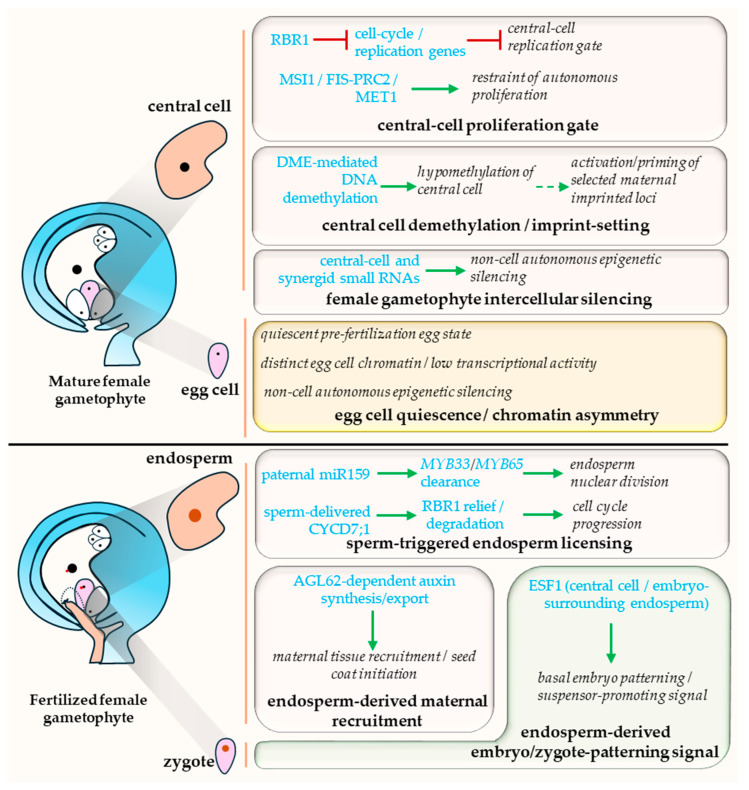
**Modular view of pre-fertilization asymmetry and fertilization-dependent release in the Arabidopsis female gametophyte.** Pre-fertilization asymmetry is shown as separable modules in the mature female gametophyte, including a central-cell proliferation gate, a DME-linked central-cell demethylation module with selective imprinting-related consequences, a small-RNA-based intercellular silencing module, and an egg-cell quiescence/chromatin-asymmetry state. After fertilization, sperm-triggered endosperm licensing is shown as the primary direct release module. Endosperm-derived maternal recruitment and endosperm-derived embryo/zygote-patterning signaling are shown separately as downstream outputs rather than as a single continuous mechanistic pathway. The primary and intermediate factors are presented in cyan texts and the effects are written in italics. Solid-colored edges indicate directly supported Arabidopsis interactions or dependencies, and dashed edges indicate indirect but supported pathway placement. The figure is qualitative and modular and does not imply a fully resolved downstream cascade.

Once the MMC has been specified, development does not proceed directly from reproductive commitment to post-fertilization proliferation. Meiosis and megagametogenesis follow to generate a mature female gametophyte in which cell identities are stabilized, proliferative capacity is actively restrained, and regulatory asymmetries relevant to later seed development are established before fertilization, although their downstream consequences are not uniform across loci and processes. This stage can appear relatively quiescent when viewed against the rapid dynamics of seed initiation, but it remains mechanistically relevant because the egg cell and central cell do not enter fertilization from equivalent chromatin, transcriptional, or cell-cycle states. These separable modules of pre-fertilization poise and their fertilization-triggered release are summarized in Figure 3.

### 3.1. Active Cell Cycle Restraint and Its Release Potential

A central feature of this stage is active pre-fertilization restraint. Early genetic evidence showed that *RETINOBLASTOMA RELATED1* (*RBR1*) is required to maintain mitotic arrest in the mature female gametophyte: in *rbr1* mutant ovules, supernumerary nuclei accumulate in the micropylar domain, and the central cell can initiate autonomous endosperm-like proliferation in the absence of fertilization [28]. Later work placed this restraint within a broader regulatory framework involving *RBR1*, *MSI1*, PRC2-associated functions, and RBR1/MSI1-linked repression of the maintenance methyltransferase *MET1* during female gametogenesis [29,30]. These studies show that the mature female gametophyte is actively maintained in a nonproliferative state before fertilization.

Recent cell-cycle analysis sharpens but does not fully settle this picture. Using DNA-sequencing-based replication profiling together with phase-specific fluorescent markers, Voichek et al. [31] argued that mature male and female gametes are arrested before DNA replication, with the central cell at G1 and the egg cell at G0, and initiate replication only during fertilization. By contrast, Simonini et al. [12] interpreted the unfertilized central cell as S-phase arrested, and the egg cell as G2-phase arrested, and proposed fertilization-dependent release through sperm-delivered CYCD7;1 and RBR1 relief in the central cell, consistent with earlier work on CYCD7;1 sperm delivery [32]. The broader handover argument does not depend on choosing definitively between these phase assignments. Across the recent literature, it is now directly supported that the mature central cell occupies an actively restrained replication-gating state before fertilization and that fertilization rapidly relieves that restraint, whereas the egg cell remains more generally quiescent and follows a distinct temporal schedule. Consistent with this, ectopic activation of CYCD7;1 is sufficient to override central-cell arrest and induce fertilization-independent proliferation [33]. Pre-fertilization quiescence is therefore an actively enforced state, and its release is not identical in the egg cell and central cell (Figure 3, proliferation gate).

### 3.2. Molecular Asymmetry and Central-Cell-Biased Imprint-Setting

The two female gametes are also molecularly unequal before fertilization. Pillot et al. [34] showed that the egg cell and central cell acquire distinct transcriptional and chromatin states during late female gametophyte development, and Ingouff et al. [35] further showed that the two fertilization products display distinct dynamics of histone H3 variants. These findings argue against a model in which embryo and endosperm arise from initially equivalent female chromatin states that diverge only after syngamy. Instead, part of their divergence is already conditioned during gametophytic maturation.

This asymmetry is especially consequential in the centra -cell, where imprinting-relevant epigenetic reprogramming is established before fertilization. *DEMETER* (*DME*) activity is required for normal imprinting and seed viability, and maternal activation of genes such as *MEA* depends on demethylation events that occur during female gametogenesis [30,36]. Park et al. [37] showed that *DME* transcription is restricted to gamete companion cells, namely the vegetative cell in pollen and the central cell in the female gametophyte, by cis-regulatory elements within the locus itself, while genome-wide methylome analysis showed that the demethylated maternal state later observed in endosperm is initiated already in the central cell before fertilization [38]. Therefore, the central cell is the best-supported site at which maternal epigenetic asymmetry relevant to later endosperm imprinting is installed before fertilization, although the downstream consequences of each pre-fertilization asymmetry are not uniform across loci (Figure 3, demethylation/imprint setting module).

Later endosperm chromatin states are consistent with this interpretation. Maternal and paternal alleles acquire distinct H3K27me3 landscapes in the endosperm, consistent with post-fertilization consequences of gamete-derived asymmetry in chromatin behavior [39]. On the paternal side, sperm chromatin is itself developmentally specialized, including large-scale resetting of H3K27me3 during sperm maturation [40]. Therefore, fertilization joins genomes that have already been differentially conditioned rather than two equivalent chromatin templates.

### 3.3. Residual Communication Within the Mature Female Gametophyte

Small-RNA behavior within the female gametophyte further supports the view that this is a poised yet still communicative structure. Earlier work on AGO9 had already implied that silencing relevant to reproductive development can be generated outside the germline proper [4]. More directly, Schroder et al. [41] showed that small RNAs can move within the female gametophyte and induce non-cell-autonomous silencing before fertilization, whereas this movement is markedly reduced afterward. Although these findings do not by themselves explain imprint establishment or cell-fate divergence, they reinforce the interpretation that the mature female gametophyte remains an integrated regulatory unit up to the point of fertilization (Figure 3, intercellular silencing module). In that limited sense, small-RNA mobility supports coordination between the egg cell and central cell state before fertilization, even though the specific downstream developmental outputs of that coordination remain unresolved.

Taken together, these modules define *poise* here in the restricted sense. The mature female gametophyte is best treated as a poised asymmetric system rather than as a passive prelude to seed development. In Arabidopsis, the best-supported components are RBR1-dependent restraint, central-cell-biased epigenetic reprogramming, and unequal female-gamete chromatin states. The broader claim that late female gametophyte development constitutes a coordinated phase of epigenetic and cell-cycle conditioning, rather than merely the completion of haploid differentiation, remains more interpretive and should be stated cautiously. Within the handover framework, this section primarily defines the prior interval-level control logic before fertilization and the asymmetries that remain relevant across the boundary; the handover claim itself is tested at fertilization.

## 4. Fertilization as Licensing Event for Seed Initiation: Release of Central-Cell Restraint and Downstream Recruitment of Maternal Tissues

Fertilization does more than unite parental genomes. Although double fertilization produces both zygote and endosperm, the clearest evidence for a fertilization-dependent release of a preassembled repressive state concerns the central cell. The embryo is considered here mainly in relation to the coordination that follows endosperm activation. In this review, licensing is therefore used narrowly for fertilization-dependent release of central-cell restraint. Broader seed-wide coordination is treated as a consequence of that event, not as part of the same mechanistic step. To keep linked but distinct processes separate, the discussion below distinguishes four levels: direct release of central-cell restraint, deployment of an endosperm-autonomous program, recruitment of maternal tissues, and subsequent embryo-endosperm coordination (Figure 3, lower panel).

### 4.1. Direct Release of Central-Cell Restraint

Not all early ovule responses require successful double fertilization. Pollen tube contents alone can trigger ovule enlargement and partial seed-coat-associated responses, showing that post-pollination activation is not a single all-or-none process [42,43]. These responses remain incomplete; however, and do not substitute for the direct activation of the central cell required for normal endosperm initiation.

Earlier work complicated any simple model in which central-cell fertilization alone explains the onset of endosperm development. Using pollen that generates only a single sperm-like cell, Nowack et al. [10] showed that fertilization of the egg cell can promote endosperm proliferation, indicating that embryo-associated or egg-fertilization-derived cues can contribute to early endosperm development. The converse case later sharpened the local logic of the transition. Using single-fertilization mutants in which the central cell is fertilized while the egg cell remains unfertilized, Xiong et al. [44] showed that endosperm initiation and much of its subsequent progression can proceed largely independently of embryogenesis, although rapid embryo expansion still accelerates later endosperm breakdown. Taken together, these studies support a restricted conclusion: embryo-endosperm coupling exists, but the decisive local trigger for normal endosperm initiation resides in the fertilized central cell.

Recent mechanistic work now supports that conclusion directly. Zhao et al. [45] showed that sperm-transmitted miR159 promotes early endosperm nuclear division by clearing central-cell-derived *MYB33* and *MYB65* transcripts after fertilization. Simonini et al. [12] further showed that sperm-delivered CYCD7;1 promotes RBR1 degradation and thereby releases the fertilized central cell into cell-cycle progression. The exact pre-fertilization cell-cycle status of the central cell remains somewhat unsettled across studies: Simonini, et al. [12] and Voichek et al. [31] differ on whether the unfertilized central cell is best described as S-phase-arrested or pre-replicative. Even so, both studies support the narrower conclusion required here. Fertilization relieves a replication-gating state in the central cell and permits rapid entry into proliferative endosperm development.

### 4.2. Endosperm-Autonomous Program

Once the restraint is released, the fertilized central cell enters an endosperm-autonomous growth program. Auxin provides the best-supported hormonal route in this transition. Figueiredo et al. [46] showed that increased auxin production after fertilization is sufficient to trigger central-cell division and is required for correct endosperm development, consistent with a model in which Polycomb-group repression blocks auxin biosynthesis before fertilization and fertilization relieves that block. Guo et al. [7] extended this framework by showing that *AGL62* is required for activation of endosperm auxin synthesis in both Arabidopsis and strawberry. Fertilization is therefore coupled to early endosperm growth through both release of central-cell restraint and induction of a self-reinforcing hormonal program within the endosperm lineage.

### 4.3. Maternal Recruitment

This endosperm output rapidly recruits maternal tissues into seed initiation. Roszak and Kohler [11] showed that Polycomb-group activity in maternal tissues represses seed-coat initiation before fertilization and that a fertilization-dependent signal from the sexual endosperm is required to release that repression. Figueiredo et al. [6] identified endosperm-derived auxin as the best-supported route for this signal and proposed that *AGL62* is required for auxin transport from the endosperm to the integuments. In parallel, Xu et al. [47] showed that nucellus degeneration is also restrained before fertilization by Polycomb-group function and depends on an *AGL62*-associated endosperm signal after fertilization. These findings place maternal-tissue recruitment downstream of central-cell release rather than within the direct licensing event itself.

### 4.4. Embryo-Endosperm Coordination

Early embryo-endosperm communication should likewise be treated as a downstream module rather than as part of the initial licensing step. Endosperm-derived ESF1 peptides influence basal embryo patterning soon after fertilization, demonstrating that communication between the two fertilization products begins very early [48]. These signals act after endosperm activation has already been initiated. The clearest local order is therefore: fertilization of the central cell, release of central-cell restraint, deployment of endosperm growth and signaling outputs, and then progressively elaborated coordination with embryo and maternal tissues.

These findings support the strongest handover claim in the present review. In Arabidopsis, fertilization converts a previously restrained central-cell state into a distinct post-fertilization control logic centered on endosperm initiation, with clear first-order consequences in the ensuing interval and substantial overlap with earlier asymmetries and restraints. Limited ovule responses can occur without successful fertilization, but normal endosperm initiation depends on direct activation of the central cell, reinforced by paternal inputs such as miR159 and CYCD7;1. The mature female gametophyte → double fertilization boundary is therefore best classified as an established handover for the central-cell-to-early-endosperm transition. Because initiation and early maintenance remain strongly overlapped and are linked by the same *AGL62*/auxin-centered logic, syncytial maintenance is treated as the ensuing interval and as a conservative, provisional assessment case rather than as a second equally independent established handover. Embryo-associated and broader seed-wide coordination are treated as downstream modules rather than as part of the core licensing event

## 5. From Syncytial Endosperm Maintenance to Cellularization: Maintenance, Exit Logic, and Developmental Consequences

**Figure 4:**
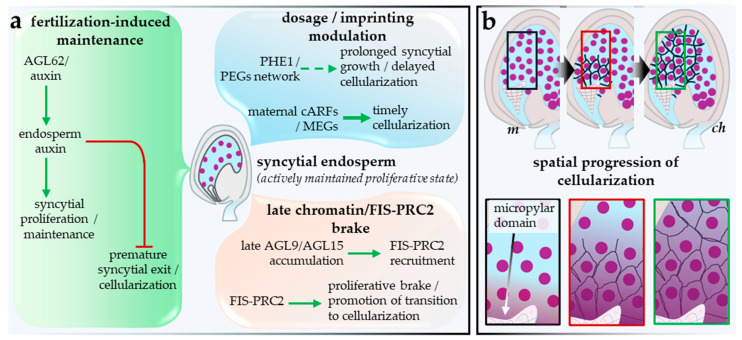
**Maintenance logic and spatial execution of endosperm cellularization in Arabidopsis.** (**a**) The syncytial endosperm is shown as an actively maintained proliferative state rather than as a passive interval before cellularization. An *AGL62*-linked endosperm auxin program induced after fertilization is depicted as the best-supported maintenance arm promoting syncytial proliferation and restraining premature syncytial exit or cellularization. Dosage/imprinting is shown separately as modulation of syncytial duration, with a PHE1/PEG-associated growth-promoting network linked to prolonged syncytial growth or delayed cellularization and a contrasting maternal cARF/MEG-associated arm favoring timely cellularization. A late chromatin/FIS-PRC2 brake is shown as an exit-promoting counterforce associated with repression of the proliferative program as the endosperm approaches transition. This partition into maintenance, modulation, and exit-promoting opposition is interpretive and is used to organize the current evidence rather than to claim a fully resolved hierarchy. (**b**) Cellularization is depicted as a spatially executed transition that begins in the micropylar domain and progresses toward the chalazal region across an already regionalized endosperm, rather than as a uniform event imposed simultaneously throughout the syncytium. This panel emphasizes observed micropylar-to-chalazal progression of partitioning of the shared syncytial cytoplasm and is intended as a developmental observation rather than a mechanistic hierarchy. Solid colored edges indicate directly supported Arabidopsis interactions or dependencies, dashed edge indicates indirect support, red inhibitory links indicate negative regulation, and black arrows indicate observed progression. In (**b**), m = micropylar end; ch = chalazal end.

The syncytial endosperm is best understood as an actively maintained interval following fertilization-dependent licensing rather than as a passive waiting period before cellularization. In Arabidopsis, proliferative control, parent-of-origin asymmetry, and interaction with maternal tissues are coordinated within this interval before the endosperm undergoes a regulated developmental transition. The argument is clearest if separated into three questions: how the syncytial state is maintained, what permits exit into cellularization, and which later seed phenotypes depend causally on cellularization rather than merely tracking alongside it. Figure 4 summarizes the core maintenance logic of the syncytial state and the spatial execution of cellularization.

The deleterious effect of both premature and failed cellularization is a central feature of this interval. Kang et al. [49] showed that *AGL62* is expressed specifically in the endosperm throughout the syncytial phase and declines abruptly just before cellularization. In *agl62* mutants, the endosperm cellularizes precociously, whereas *AGL62* expression persists and cellularization fails in mutants defective in *FERTILIZATION-INDEPENDENT SEED* (*FIS*) Polycomb-group function. These results established *AGL62* as a key suppressor of premature cellularization and suggested that FIS-PRC2 promotes the syncytial-to-cellular transition, directly or indirectly, through timely repression of *AGL62*. Figueiredo et al. [46] then linked this developmental switch directly to auxin by showing that elevated auxin production after fertilization is sufficient to promote endosperm proliferation and prevent premature cellularization. Guo et al. [7] further placed *AGL62* upstream of fertilization-induced endosperm auxin synthesis in both Arabidopsis and strawberry. Batista et al. [50] strengthens this framework at the network level by identifying *PHERES1* (*PHE1*) as a central regulator of endosperm transcriptional circuitry that includes auxin-related targets such as *YUC10*. Together, these studies support a restrained but strong conclusion: the syncytial state is actively maintained through an *AGL62*- and auxin-linked control logic, and cellularization represents regulated exit from that logic rather than a passive endpoint of proliferation or a purely morphological transition (Figure 4a).

That control logic is also highly dosage sensitive because endosperm development is intrinsically parent-of-origin asymmetric. Kradolfer et al. [51] showed that increasing maternal genome dosage can bypass the requirement for FIS-PRC2 in seed development, suggesting that one important function of the complex is to buffer dosage-dependent growth imbalance in the endosperm. Batista et al. [50] subsequently reported *PHE1* as a major regulator of paternally biased endosperm gene expression and of growth-promoting downstream targets, placing paternalized dosage logic within the broader regulatory landscape that influences syncytial maintenance. Wolff et al. [52] then showed that paternal loss of *PEG2*, *PEG9*, or *SUVH7* restores endosperm cellularization and substantially rescues triploid seed viability, demonstrating that a subset of paternally expressed imprinted genes can act genetically upstream of persistent syncytial maintenance in paternal-excess seeds. Their transcriptomic analysis further suggested that these rescue effects converge on downstream normalization of cell-wall-remodeling programs, including pectin-degradation-linked expression. Recent work has also clarified the maternal counterweight to this circuitry. Butel et al. [53] identified a clustered family of maternally expressed, paternally silenced auxin response factors that promote endosperm cellularization while restricting seed growth. Therefore, parental asymmetry defines a dosage-sensitive control layer that influences how long the syncytial program is maintained and how permissive the system becomes for cellularization, rather than resolving a single trigger hierarchy (Figure 4a, dosage/imprinting axis).

Late syncytial development also involves an active chromatin-based braking system. Weinhofer et al. [54] showed that the endosperm H3K27me3 landscape includes targets with functions relevant to cellularization and chromatin architecture, and Moreno-Romero et al. [39] later defined strong parental asymmetry in PRC2-mediated chromatin states in the endosperm. Most directly, Zhang et al. [55] showed that AGL9 and AGL15 accumulate toward the end of syncytial development and recruit the FIS-PRC2 complex to key proliferation loci such as *IKU2* and *MINI3*, thereby promoting deposition of repressive chromatin marks as the endosperm exits its proliferative state. These data support a late transcriptional and chromatin program that helps terminate syncytial proliferation. However, at present, the evidence is stronger for partially linked exit modules than for one ordered master switch spanning *AGL62* decline, auxin dynamics, dosage-sensitive imprinting circuitry, and late PRC2 recruitment (Figure 4a, late chromatin brake).

Cellularization is also spatially patterned rather than uniform. In Arabidopsis, it begins in the micropylar region and progresses across an already regionalized endosperm, indicating that the transition is executed within a differentiated tissue rather than imposed simultaneously by a single clock-like trigger [15]. The transition also changes what the endosperm can do: as a syncytium, it supports rapid expansion and extensive cytoplasmic continuity, whereas after cellularization, it becomes progressively partitioned and functionally restricted (Figure 4b).

**Figure 5:**
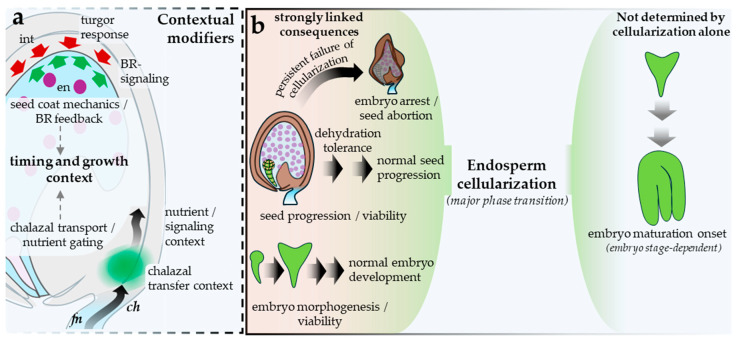
**Contextual modifiers and developmental consequences of endosperm cellularization in Arabidopsis.** (**a**) Mechanical, maternal, and transport-related influences are shown as contextual modifiers rather than established primary triggers of endosperm cellularization. Seed coat mechanics and BR-linked feedback are presented as shaping the timing and growth context of transition, whereas chalazal transport and nutrient/signaling context are shown as broader physiological inputs. These features are therefore placed outside the core exit logic and given weaker visual weight. (**b**) Developmental consequences of endosperm cellularization are separated into outcomes strongly linked to successful or failed transition and outcomes not determined by cellularization alone. Persistent failure of cellularization is strongly associated with embryo arrest and seed abortion, whereas successful progression is linked to normal seed progression, embryo morphogenesis and viability, and dehydration tolerance associated with seed survival. By contrast, embryo maturation onset is shown as embryo stage-dependent rather than as a direct output of cellularization itself. Dashed panel borders mark inferred framing, dotted gray elements indicate contextual synthesis, and black arrows indicate schematic developmental progression or association rather than a fully resolved causal chain.

Several additional processes shape the developmental setting of syncytial exit but are better treated as contextual modifiers than as core triggers of cellularization. Beauzamy et al. [56] showed that early whole-seed turgor is generated largely by the endosperm and declines as cellularization and growth arrest approach. Creff et al. [57] extended this view by showing that endosperm pressure has antagonistic effects: it promotes seed expansion directly, yet also restricts further growth indirectly by increasing seed-coat tension and promoting wall stiffening. Lima et al. [8] provide a maternal counterpart, showing that brassinosteroid signaling in the seed coat feeds back on endosperm proliferation through maternal biophysical properties rather than through the timing of cellularization itself. Liu et al. [9] further identified a fertilization-dependent phloem-terminal gate in the chalazal region whose opening increases nutrient flow into the developing seed. These inputs are therefore treated here as modifiers of cellularization timing and growth setting, not as established components of the core exit logic (Figure 5a).

Persistent failure of endosperm cellularization compromises seed progression [15], but its downstream consequences are not uniform. The strongest evidence links cellularization to viability, embryo morphogenesis, and embryo-endosperm coordination, whereas embryo maturation onset depends more directly on embryo developmental stage than on cellularization *per se* [58]. Xu et al. [59] sharpened this point mechanistically by showing that embryos enclosed by uncellularized endosperm mount an osmotic-stress response with elevated ABA signaling, consistent with the view that cellularization contributes to the dehydration tolerance required for survival through seed maturation. The downstream consequences of successful or failed cellularization, including outcomes that are strongly linked versus those not determined by cellularization alone, are separated in Figure 5b.

Endosperm-derived signals help explain why cellularization can matter strongly without serving as a universal instructor. ESF1 peptides produced from the central cell and the embryo-surrounding endosperm influence early embryo patterning [48], and endosperm-expressed *LEC1* is required for normal embryo maturation in Arabidopsis [60]. These findings show that the endosperm can act instructively, but the signals identified so far arise from specific endosperm domains and transcriptional states rather than from cellularization alone. At the same time, embryo progression is not simply dictated from outside. Simonini et al. [61] showed that *MEDEA* (*MEA*) acts within the embryo to control proliferation and patterning through repression of *CYCD1;1*, indicating that embryonic competence remains under strong intrinsic control.

Overall, Arabidopsis evidence supports treating the syncytial endosperm as an actively regulated developmental interval rather than a purely morphological one. *AGL62*- and auxin-linked maintenance, dosage-sensitive parental asymmetry, and late PRC2-associated chromatin braking all influence when this prior control logic can be terminated, while mechanics and nutrient access act best as contextual modifiers of the setting in which the transition occurs. By the framework used here, the syncytial endosperm → endosperm cellularization boundary remains a provisional handover: the maintained prior control logic is clear, and crossing into the ensuing interval has detectable first-order consequences, but the new principal organizer of progression and the ordering of exit remain only partly resolved. Cellularization is therefore better treated as a major gate in seed progression than as a universal determinant of embryo development (Table 2). Figure 5 summarizes the principal contextual modifiers and downstream consequence channels discussed here; these are included to delimit the biological significance and limits of the cellularization transition rather than to propose an additional handover boundary.

## 6. How Far Does the Arabidopsis-Centered Handover Model Generalize?

The answer depends on the level of claim being made. Rather than to export Arabidopsis pathway architecture wholesale, the aim here is to distinguish among three different kinds of comparison: direct mechanistic extension beyond Arabidopsis, recurrence of a broader developmental problem or regulatory logic, and lineage-specific solutions that mark the limits of generalization. Because Arabidopsis provides the most complete mechanistic resolution for the interval considered here, cross-species extension is most defensible when made claim by claim rather than by treating the full handover framework as already universal. Where support is strongest only in Arabidopsis or remains review-level outside that system, that limitation is stated explicitly [48,60,62,63].

At the pre-fertilization end, direct mechanistic extension remains limited. Small-RNA restriction, RNA-directed DNA methylation, KLU-mediated chromatin control, and the ARF17-SPL/NZZ auxin module are best treated as Arabidopsis-resolved mechanisms rather than demonstrated angiosperm-wide machinery. Broadly concluded, the female reproductive fate is established within a somatic ovule context rather than as an isolated cell-autonomous event. A similar distinction applies after meiosis. Unequal regulatory states between female gametes are likely to be broadly relevant, but direct comparative support remains strongest in Arabidopsis. In rice, the clearest primary anchor is that the maternal hypomethylated state later observed in endosperm is already initiated in the central cell before fertilization [38]. Pre-fertilization asymmetry can therefore be generalized more confidently than any specific upstream Arabidopsis mechanism.

Post-fertilization comparisons are stronger at the level of developmental organization or process than at the level of shared pathway architecture. In Arabidopsis and other systems examined, early seed development depends on coordination among embryo, endosperm, and maternal tissues rather than on isolated progression of each compartment, but the mechanistic basis of that coordination is not equally resolved across angiosperms [48,60,62]. One important direct extension beyond Arabidopsis does exist: *AGL62*-dependent activation of endosperm auxin synthesis has been shown in both Arabidopsis and strawberry, although the downstream architectures are not identical [7]. Beyond that, the strongest comparisons concern recurring developmental logic rather than the shared pathway architecture. By contrast, sperm-delivered miR159 and CYCD7;1 should still be treated as Arabidopsis-resolved mechanisms rather than broadly established angiosperm triggers [12,45]. The transferable claim, then, is that fertilization can release a restrained endosperm program and recruit broader seed coordination, whereas the specific triggering modules remain partly lineage- or system-specific.

The least portable part of the framework concerns endosperm developmental form and dosage circuitry. Endosperm ontogeny varies widely across angiosperms, and even among coenocytic systems, syncytial duration, cellularization timing, and the developmental meaning of cellularization differ substantially. The Arabidopsis sequence of syncytial proliferation followed by cellularization should therefore not be treated as a canonical universal program [14,16]. More secure is the inference that endosperm transition control poses a recurrence of a dosage-sensitive problem. Rice provides the strongest non-Arabidopsis mechanistic anchor for this process-level recurrence: *OsEMF2a* is required for endosperm cellularization and imprinting, *osemf2a* mutants show autonomous endosperm development together with delayed post-fertilization cellularization, and increasing maternal ploidy can rescue interspecific endosperm failure while restoring cellularization timing, nucellus degeneration, storage-product accumulation, and imprinted-gene expression [64,65,66]. These findings support recurrence of a PRC2-linked, dosage-sensitive endosperm transition problem without implying one-to-one conservation of Arabidopsis FIS-PRC2 circuitry.

Comparable endosperm-centered barriers recur across eudicots, but these examples are most informative as evidence of a recurring developmental problem coupled to lineage-specific solutions, rather than of conservation of a single Arabidopsis downstream network. In *Capsella*, reciprocal hybrids fail through opposite endosperm cellularization syndromes that resemble paternal- and maternal-excess outcomes [67]. In *Mimulus* and wild tomatoes, hybrid seed inviability again localizes to early endosperm malfunction, although the visible route is impaired endosperm development or proliferation rather than an Arabidopsis-like syncytial-delay phenotype [68,69]. Dosage imbalance, imprinting, and epigenetic asymmetry therefore recur more broadly than any single Arabidopsis downstream network, and lineage-specific mechanisms such as maternal siRNA dosage effects in *Capsella* should be stated as such rather than generalized across angiosperms [50,70,71,72,73].

**Table 2:** **Operational assessment of proposed developmental handovers from MMC specification to endosperm cellularization.** Classification refers to handover status; evidence grade refers to the strength of Arabidopsis support for the criterion-level inference at each boundary. Detailed anchor-by-anchor criterion assignments are provided in intentionally more granular Table S1.

Boundary Assessed	Classification	Criteria Assessment (C1–C4)	Evidence-Calibrated Synthesis Statement	Representative Primary Anchors (Arabidopsis Emphasis)	Evidence Grade and Main Limiting Condition
**MMC specification → mature female gametophyte** (composite pre-fertilization span)	**Provisional** (composite assessment case; not event-centered)	**C1 yes:** An ovule-context control logic of sporophytic restriction, together with localized competence around MMC selection, is identifiable. **C2 partial:** An ensuing mature female-gametophytic poise state is identifiable, but emergence of that state as the new principal organizer across the intervening pre-fertilization span is not boundary-localized. **C3 yes:** Detectable first-order consequences of traversing this broader pre-fertilization span are present in the ensuing interval, which is organized as an unequal, actively restrained mature female gametophyte. **C4 yes (contextual/synthesis-level):** Overlap between earlier MMC-centered restriction/selection logic and later gametophytic conditioning is supported mainly at the synthesis level, with only limited study-level anchors rather than a single direct boundary-localized demonstration.	Arabidopsis supports MMC singleness and mature female-gametophytic poise as biologically real states within one broader pre-fertilization span. The ensuing poise state and its detectable downstream asymmetries are clear, but the dominant shift in control linking MMC restriction to that later interval is not yet directly resolved across meiosis and early gametophytic differentiation. For this reason, the boundary remains provisional, and overlap across the span is best treated as contextual and synthesis-level rather than as a single boundary-localized proof.	**C1 prior logic:** Olmedo-Monfil et al. [4]; Rodriguez-Leal et al. [20]; Mendes et al. [3]; Zhao et al. [18]; Cao et al. [21] **C2 ensuing poise state (partial):** Jullien et al. [30]; Pillot et al. [34]; Park et al. [38]; Voichek et al. [31] **C3 detectable ensuing-interval consequences:** Jullien et al. [30]; Pillot et al. [34]; Park et al. [38]; Voichek et al. [31] **C4 overlap (contextual/synthesis-level):** Mendes et al. [3]; Pillot et al. [34]; synthesis across the full pre-fertilization evidence base	Strong for MMC singleness and mature female-gametophytic poise when considered separately; moderate for treating the intervening pre-fertilization span as a single assessed handover unit. Main limit: C2 remains only partially resolved, because the dominant control shift linking MMC restriction to later female-gametophytic poise is not yet localized as a single boundary-level transfer, while overlap is supported mainly contextually rather than by one direct anchor.
**Mature female gametophyte → double fertilization**	**Established** handover	**C1 yes:** A prior control logic of pre-fertilization central-cell restraint and gamete asymmetry is directly supported. **C2 yes:** Fertilization-dependent release of central-cell restraint and initiation of endosperm development become the dominant organizer of progression. **C3 yes:** Crossing this boundary has detectable first-order consequences in the ensuing interval, including endosperm initiation and early maternal recruitment outputs. **C4 yes:** Pre-fertilization asymmetry overlaps with early post-fertilization lineage-specific and imprint-related behavior rather than being erased at the boundary.	The clearest Arabidopsis handover occurs when fertilization relieves central-cell restraint and fertilization-dependent endosperm initiation becomes the dominant organizer of early seed progression. This is a boundary-localized shift in control, even though the internal ordering among paternal cargos, central-cell-autonomous release, and earliest downstream relays remains incompletely resolved.	**C1 prior control logic:** Ebel et al. [28]; Jullien et al. [30]; Sornay et al. [33]; Pillot et al. [34] **C2 new organizer:** Zhao et al. [45]; Simonini et al. [12]; Figueiredo et al. [46]; Guo et al. [7] **C3 detectable ensuing consequences:** Roszak and Kohler [11]; Figueiredo et al. [6]; Xu et al. [47] **C4 overlap:** Jullien et al. [30]; Pillot et al. [34]; Figueiredo et al. [6]	Strong. Main limit: the internal ordering among paternal cargos, central-cell-autonomous release, and earliest downstream relays remains incompletely resolved, but this does not overturn the established boundary-level classification.
**Double fertilization → syncytial endosperm**	**Provisional** handover (overlap-heavy)	**C1 yes:** the prior control logic of fertilization-dependent licensing and endosperm initiation is identifiable. **C2 partial:** a maintained syncytial program clearly emerges, but its onset is not cleanly separable from the established licensing transition. **C3 partial:** crossing into the ensuing interval has detectable first-order consequences, including sustained syncytial proliferation and dosage-sensitive elaboration of maintenance, but these remain too continuous with the licensing logic to support a distinct second handover. **C4 yes:** paternal inputs, maternal recruitment, and early syncytial maintenance logic overlap substantially across this boundary.	Arabidopsis strongly supports an actively maintained syncytial program after fertilization. This row is retained as a conservative assessment case, but the onset of that maintained interval remains too overlapped with fertilization-dependent licensing to justify a second equally independent established handover. Detectable consequences in the ensuing interval are present, but they are not discretely separable enough from the licensing transition to elevate this boundary beyond provisional status.	**C1 prior control logic:** Zhao et al. [45]; Simonini et al. [12]; Figueiredo et al. [46] **C2 ensuing maintained program:** Kang et al. [49]; Figueiredo et al. [46]; Guo et al. [7] **C3 detectable ensuing consequences (partial):** Batista et al. [50]; Kradolfer et al. [51]; Butel et al. [53] **C4 overlap:** Roszak and Kohler [11]; Figueiredo et al. [6]; Xu et al. [47]	Strong for the existence of an actively maintained syncytial program; Moderate for treating its onset as a distinct handover. Main limit: the same AGL62/auxin-centered logic spans initiation and early maintenance, so criterion C2 is not cleanly separable from the preceding established handover.
**Syncytial endosperm → endosperm cellularization**	**Provisional** handover	**C1 yes:** The maintained syncytial control logic and its major sustaining inputs are identifiable. **C2 partial:** Exit to cellularization is clear, but no single dominant organizer of the transition is fully resolved. **C3 partial:** Crossing this boundary has detectable first-order consequences, including spatially patterned cellularization and immediate changes in seed progression. **C4 yes:** Auxin-linked maintenance, dosage-sensitive control, and late chromatin inputs overlap substantially across exit.	Arabidopsis supports a real developmental transition out of syncytial proliferation, but the boundary remains provisional because cellularization appears to arise from convergence of attenuated maintenance, dosage-sensitive regulation, late chromatin braking, and spatial execution rather than from one fully resolved trigger hierarchy.	**C1 prior control logic:** Kang et al. [49]; Figueiredo et al. [46]; Guo et al. [7] **C2 integrated exit logic:** Wolff et al. [52]; Batista et al. [50]; Butel et al. [53]; Zhang et al. [55] **C3 detectable ensuing consequences:** Hehenberger et al. [15]; Xu et al. [59] **C4 overlap:** Weinhofer et al. [54]; Moreno-Romero et al. [39]; Zhang et al. [55]	Strong for patterned cellularization and its developmental importance; Moderate for integrated exit logic. Main limit: criterion C2 is not yet met cleanly because no single dominant organizer of the exit transition has been resolved.

**Table note:** Boundary labels identify stage changes; regime terms in the synthesis statements refer to the ensuing interval. Established handover denotes a boundary for which all four criteria are directly supported in Arabidopsis. Provisional handover denotes a biologically plausible shift in control for which one or more criteria remain partly unresolved. C1 = dominant control logic before the boundary is identifiable; C2 = distinct dominant control logic after the boundary is identifiable; C3 = crossing the boundary has detectable first-order consequences in the ensuing interval; C4 = earlier and later control logics or influences remain partially overlapping across the boundary. For C1–C4, yes = direct Arabidopsis support for the criterion as framed at that boundary; partial = biologically plausible but incompletely resolved dominance, ordering, or boundary localization; no = criterion not currently met. Evidence grade and handover classification are assigned separately. Representative anchor sets are selective rather than exhaustive.

The same scoping principle applies to embryo dependence. Current comparative evidence supports the broad conclusion that endosperm state conditions embryo development, but not a universal checkpoint relationship between the two compartments [48,60,74]. In cereals, the endosperm has a more extended nutritive and signaling role because of its prolonged persistence and stronger compartmentalization. Maize provides a concrete primary anchor: a discrete embryo-facing endosperm subdomain adjacent to the scutellum is enriched for transporter-related functions and responds to embryo development, while earlier seed transcriptomics already showed strong embryo-endosperm transcriptional divergence [75,76]. This comparison is therefore best read as evidence of lineage divergence in how embryo-endosperm coordination is organized. What generalizes more securely is the recurrence of embryo-facing endosperm interface specialization, not any single Arabidopsis signaling module.

Taken together, the Arabidopsis-centered handover framework generalizes best at the level of staged restraint, release, and cross-compartment coordination. It generalizes less securely at the level of specific regulators, endosperm developmental form, or the assumption that syncytial proliferation followed by cellularization defines a standard angiosperm sequence. Used in this restricted way, the framework is not a claim of universal pathway conservation, but a comparative template for distinguishing what is directly portable, what recurs as a shared developmental problem, and what remains lineage-contingent.

## 7. Outstanding Questions and Future Directions for a Restraint-to-Release Model

A handover framework is useful only insofar as it shows where the four defined criteria are met and where they remain unresolved. The most informative next experiments will therefore be those that discriminate the criteria directly, rather than simply add regulators to already crowded models. Early seed development clearly depends on sustained communication among embryo, endosperm, and maternal tissues rather than on the isolated progression of each compartment [6,11,13,77]. The central experimental priority is to determine which boundaries truly involve a change in dominant control and which instead reflect redeployment of similar control logic in a new developmental setting.

For the MMC interval, C2 remains the main unresolved criterion, whereas C3 is treated as sufficiently supported for classification purposes but remains open to sharper mechanistic localization. In Arabidopsis, the evidence strongly supports a model in which sporophytic restriction, local competence, chromatin remodeling, DNA methylation balance, and auxin responsiveness all contribute to MMC singleness [2,3,4,17,18]. However, the order in which these asymmetries arise, and the extent to which some stabilize rather than initiate MMC selection, remain unclear. It is not yet resolved whether chromatin asymmetry first biases auxin responsiveness and SPL/NZZ expression, whether positional signaling initiates epigenetic divergence of the future MMC, or whether both processes are coupled from the outset [2,17,26]. The possible contribution of ovule biophysics belongs within this unresolved space rather than outside the main argument: primordium growth, tissue geometry, and mechanical context may influence where reproductive competence emerges, but that possibility remains much less directly tested than the small-RNA, methylation, and chromatin pathways discussed above. The most useful next experiments are therefore the tests that discriminate C2 from C3: does perturbing one candidate upstream module reassign the dominant control logic of the boundary itself, or does it mainly alter the molecular state of the ensuing female-gametophytic interval while leaving the broader boundary architecture intact?

For the mature female gametophyte-to-fertilization boundary, C1 to C4 are sufficiently supported for classification purposes, but the mechanistic ordering within that established boundary remains incompletely resolved. Current evidence supports active restraint in the mature female gametophyte, unequal chromatin states in egg and central cell, and fertilization-triggered release mechanisms that are especially clear in the central-cell lineage [12,31,34,37,40]. However, it remains unclear how female-gamete conditioning, paternal cargo, and fertilization-dependent signaling are integrated to produce rapid endosperm activation while the zygote follows a different developmental schedule. A related question is whether the onset of syncytial maintenance should be treated as a second boundary-level handover or more conservatively as the early interval-level continuation of the fertilization-dependent release event. That issue is best framed as a discriminator of C2 and C3: do early post-fertilization inputs define a distinct new principal control logic after licensing, or do they mainly elaborate first-order consequences within the same boundary crossing? Next key step is to determine which paternal or fertilization-dependent inputs are necessary, which are redundant, which act at the licensing boundary itself, and which instead sustain the ensuing syncytial interval or represent Arabidopsis-specific implementations rather than broadly conserved angiosperm mechanisms [12,45].

For the syncytial-to-cellularization boundary, the main missing criterion is again C2: the local biology is increasingly well supported, but the new principal organizer of progression is still not identified with confidence. C3 is treated as sufficiently supported for classification purposes because downstream first-order consequences of exit are clearly detectable, even though no single upstream exit-promoting logic has yet been mechanistically localized as decisive. The literature now supports a multi-input model involving AGL62, auxin, dosage-sensitive parental asymmetry, and late chromatin-based repression [7,49,50,51,53,55]. Recent work also indicates that maternal tissues, mechanics, and nutrient access shape the context in which this transition occurs [8,9,56,57]. However, current evidence does not yet distinguish whether cellularization begins when a hormonal threshold is crossed, when proliferative chromatin states are sufficiently attenuated, when mechanical or nutritional conditions change, or only when several thresholds coincide. The experiments on this boundary should be framed to discriminate C2 from C3 directly: which perturbations change what organizes progression across the boundary, and which instead alter only the detectable downstream consequences of exit once the transition has already begun? Downstream seed and embryo responses after cellularization remain biologically important, but they are treated here as heterogeneous consequence channels beyond the handover series considered here, rather than as a separate post-cellularization boundary.

Comparative testing should likewise ask which handover criteria transfer across species and which do not, rather than only asking whether named regulators can be found outside Arabidopsis. Broad conservation of maternal-filial coordination, dosage sensitivity, and endosperm-mediated signaling is already supported, but substantial diversity remains in endosperm persistence, transfer structures, imprinting landscapes, and the developmental meaning of cellularization across angiosperms [7,13,14,16,38,78]. Next informative studies need to pair Arabidopsis-style mechanistic resolution with matched datasets from systems that retain persistent endosperm, elaborate transfer domains, or divergent parent-of-origin programs.

Three experimental outcomes would most clearly discriminate a true handover model from a repeated-logic model. First, perturbing an upstream restraining logic should measurably alter the molecular or physiological state of the ensuing interval, even when gross stage identity is preserved; this would provide the clearest discriminator of C3 by asking whether first-order boundary consequences are specifically altered rather than merely accompanied by broader interval disruption. Second, experimentally uncoupling central-cell replication gating, fertilization-dependent endosperm activation, syncytial auxin output, and maternal-tissue recruitment should reveal which components belong to the licensing boundary itself and which instead sustain the ensuing interval; this would sharpen C2 most directly while also clarifying C4 by distinguishing a new principal control logic from overlapping downstream modules. Third, shifting cellularization timing independently of mechanics or nutrient flux would clarify whether cellularization is best interpreted as a local endosperm switch, a seed-wide systems transition, or both; this would most directly test C2 by identifying what actually organizes progression across the exit boundary.

These questions underscore the main value of the framework. It should function as an experimental bookkeeping of where dominant control logics truly change, what overlap persists, and which first-order consequences emerge in the ensuing interval, rather than as a finished explanation. Progress will depend on integrating genetics, live imaging, biomechanics, and comparative developmental analysis rather than treating these as separate levels of explanation.

## 8. Conclusions

Early seed initiation in Arabidopsis is best read as a developmental continuum (Figure 1). Nevertheless, only one boundary currently satisfies the handover criteria strongly: the fertilization-dependent release of central-cell restraint. The MMC specification → mature female gametophyte, double fertilization → syncytial endosperm, and syncytial endosperm → endosperm cellularization boundaries remain provisional mainly because criterion 2, the emergence of a distinct dominant control logic in the ensuing interval, remains incompletely resolved, even where the biology of the intervals themselves is well supported (Table 2). The double fertilization → syncytial endosperm interval is therefore retained more conservatively as a maintained consequence of fertilization-dependent release than as a second equally independent established handover. The handover series considered here concludes at cellularization, whereas post-cellularization seed and embryo responses are treated as heterogeneous downstream consequences rather than as a further handover.

Therefore, the handover framework makes stage boundaries experimentally assessable without replacing canonical descriptive staging. Its value lies in asking where dominant control logics truly change, what overlap persists across boundaries, and which first-order consequences emerge in the ensuing interval. Arabidopsis remains the best system in which to resolve those questions, and comparative work should test boundary logic rather than merely transport named regulators across species.

## Figures and Tables

**Table 1:** Roadmap of early Arabidopsis seed-initiation transitions and interval logic.

Reviewed Transition/Roadmap Step	Dominant Interval-Level Control Logic	Main Emphasis in the Manuscript	Principal Tissues/Compartments	Handover Status in This Review
Ovule primordia → MMC specification	Ovule patterning/sporophytic restriction	Primordium context and restriction of supernumerary MMC-like fate.	Ovule primordium, nucellus, sporophytic tissues	Context lead-in (setup for later boundary analysis)
MMC specification → mature female gametophyte	Composite pre-fertilization trajectory: MMC restriction to female-gametophytic poise	MMC singleness and later mature female-gametophytic poise are treated as the linked ends of one broad pre-fertilization assessment unit, rather than as a single clean boundary-localized transfer.	MMC, developing female gametophyte, egg cell, central cell	Provisional composite assessment unit (not event-centered; the ensuing poise state is clear, but the dominant control shift across the intervening pre-fertilization span remains incompletely boundary-localized)
Mature female gametophyte → double fertilization	Fertilization-dependent licensing	Boundary-localized release of central-cell restraint and initiation of endosperm development.	Central cell/endosperm, egg/zygote, maternal interface	Established (clearest boundary-localized release handover)
Double fertilization → syncytial endosperm	Syncytial maintenance (overlap-heavy ensuing interval)	Emergence of an actively maintained syncytial interval after fertilization-dependent licensing.	Syncytial endosperm, maternal tissues, zygote/embryo	Provisional (strongly supported ensuing interval; onset remains strongly overlapped with the preceding licensing transition)
Syncytial endosperm → endosperm cellularization	Cellularization exit logic/phase transition	Regulated exit from the syncytial state with dosage-sensitive modulation, late chromatin braking, and spatial execution.	Endosperm, embryo, maternal tissues	Provisional (major transition; exit is real, but no single principal organizer or trigger order is fully resolved)

Note: Table 1 mixes roadmap steps and candidate handover units. Only transitions that can be evaluated as candidate shifts in dominant control are treated as handover assessments; broader developmental spans are retained as composite roadmap units when boundary-level localization remains incomplete. A fuller boundary-by-boundary assessment is provided in Table 2.

## Data Availability

No new data were created or analyzed in this study.

## Supplementary Materials

The following supporting information can be downloaded at: https://www.mdpi.com/article/10.3390/plants15091410/s1. Table S1: Criterion-level anchor details defined in Table 2.
